# Behaviour of Knitted Materials in a Vibrating Environment

**DOI:** 10.3390/ma18030479

**Published:** 2025-01-21

**Authors:** Mirela Blaga, Neculai Eugen Seghedin, Mihăiță Horodincă, Cristina Grosu, Hassen Gaaloul, Amel Babay, Soufien Dhouib, Bechir Azouz

**Affiliations:** 1 Faculty of Industrial Design and Business Management, “Gheorghe Asachi” Technical University of Iasi, 700050 Iasi, Romania; cristina.grosu@student.tuiasi.ro; 2 Faculty of Machine Manufacturing and Industrial Management, “Gheorghe Asachi” Technical University of Iasi, 700050 Iasi, Romania; neculai-eugen.seghedin@academic.tuiasi.ro (N.E.S.); mihaita.horodinca@academic.tuiasi.ro (M.H.); 3 Textile Engineering Department, ISET of Ksar Hellal, Ksar Hellal, Monastir 5019, Tunisia; hassengaaloul90@gmail.com (H.G.); babay_amel@yahoo.fr (A.B.); soufien_dhouib@yahoo.fr (S.D.); azzouzbechiir@yahoo.fr (B.A.); 4 Monastir University, Textile Engineering Laboratory, LR11ES42, Ksar Hellal, Monastir 5019, Tunisia

**Keywords:** weft-knitted spacer fabrics, natural frequencies, vibration transmissibility

## Abstract

The energy generated by the impact of vibrations from industrial tools or ongoing activities can be transmitted to humans and cause various injuries. Knitted materials can be considered as parts of anti-vibration equipment as they have proven their ability to absorb shocks. In this study, six spacer knitted fabrics consisting of two outer layers of cotton yarns (Nm 1/50 and Nm 1/40) and cashmere yarns (Nm 2/56) connected by PES monofilaments with a diameter of 0.08 mm were tested. To date, the use of natural yarns in the outer layers of spacer fabrics used in environments subject to vibration has been less studied. The first part of the experiments deals with the measurement of the natural frequencies of the materials, which were determined using the free vibration method. The results show that the direction of the experiment, the yarn count, the stitch density, and the thickness of the material influence the value of the natural frequencies. These values are relevant in order to avoid undesirable resonances that occur when the excitation frequency of an external system overlaps with the natural frequency of the material. In the second part, the vibration transmissibility was simulated using a vibration system with one degree of freedom. The fabrics composed of cotton yarns Nm 1/50 had the highest damping capacity and the highest specific damping coefficient and the lowest value for vibration transmission, which make them recommendable for protective materials.

## 1. Introduction

Workers who operate instruments like asphalt hammers, riveting guns, demolition picks, and either fixed or mobile equipment like dumpers, scrapers, or excavators are exposed to hand-arm vibration, foot-transmitted vibration, or whole-body vibration phenomena, causing permanent occupational diseases, such as disorders of the circulatory system, sensory nerves, muscles, bones, and joints of the hands and arms [1,2,3,4,5]. The damage caused by excessive exposure is significantly related to the level of vibrations produced by the tool, with the daily exposure limits being imposed through the European framework [6]. Besides the framework, a strict policy in terms of preventing and reducing exposure risks is mandatory by establishing proper work equipment selection, personal protective equipment (PPE) usage and time, and intensity of exposure limitations [7,8]. To meet all of these health and safety requirements regarding the exposure of workers to the risks arising from vibration, it is necessary to study the vibration behaviour of the damping materials. In this context, knowing the values of the natural frequencies of materials becomes relevant; otherwise, resonance may occur, a dangerous phenomenon that happens when the excitation frequency of an external force overlaps with the natural frequency of the system [9].

Knitted materials were recently explored as damping layers, as air bladders, foam, rubber, or polymer gels, abundantly used in PPE construction in recent decades [10,11,12,13], compromise dexterity and sense of touch and reduce comfort because of the low values of air permeability and moisture transmission [14].

Spacer knitted fabrics are complex three-dimensional structures obtained by knitting two outer layers connected by yarns or other knitted layers in different bond ratios. Their valuable performances, like high compressibility, breathability, impact resistance, elasticity, and elastic recovery, were highlighted through a series of research studies by analysing the spacer fabric characteristics and parameters with the most influence on the behaviour in a vibration environment, such as spacer yarn threading, spacer yarn diameter, yarn consumption, fabric thickness, and raw material [15,16,17,18]. Recent tests on spacer fabrics specifically designed for vehicle driver seats showed good vibration isolation in the frequency range of 0–35 Hz, as it is known that low and medium frequencies have the strongest negative effects on human health [19,20,21]. Different natures of raw materials were used for the outer layers: natural, synthetic, artificial, or high-performance fibres, even for some patented gloves [22]; however, very few studies have evaluated the impact of yarn types on the ability to reduce vibration transmission [23]. The authors observed that the response of the fabrics in dynamic tests is correlated to the yarn type, revealing significant differences between the frequencies of fabrics created using synthetic and natural yarns [24].

The vibration behaviour and physical properties of spacer fabrics produced by flat knitting technology showed that to extend the frequency range for vibration isolation, spacer fabrics with smaller monofilament diameter and longer linking distance were preferred. A nylon monofilament was preferred over a polyester monofilament to achieve optimal fabric thickness. A higher monofilament diameter resulted in a lower stitch density and a higher mass per unit area of the spacer fabric [25]. The fabric with a polyamide monofilament as a spacer thread in the middle layer generated more internal resonance than the one with a polyester monofilament. The insertion of hollow silicone tubes increases the degree of damping vibration isolation, while inserted silicone foam tubes have the opposite effect [26]. A free vibration test was performed, and the corresponding model was built based on a system with one degree of freedom (SDOF), taking into account the viscoelastic behaviour of the warp knitted spacer. The experimental results of the damping ratio and displacement–time curves were compared with the theoretical results. It was shown that the vibration model can characterise the free vibration of spacer fabrics well [17,27,28].

In addition to mechanical performance, the comfort performance of anti-vibration knitted fabrics should also be taken into account, as cold temperature is the main factor that increases the negative effects of vibrations on the human body. Therefore, special attention must be paid to the selected raw materials in order to minimise these risks, starting with the analysis of the materials used so far. Natural fibres such as cotton are often used for anti-vibration gloves [10]. Synthetic fibres are the most commonly used raw material for knitted anti-vibration structures. Their damping performance has been studied in numerous experiments, but the influence of the raw material on the vibration behaviour has only rarely been analysed [22,29,30,31]. High-performance fibres such as Kevlar or Dyneema have been used in the development of anti-vibration gloves for specific applications where cut-resistant and fire-resistant properties are required. In some models of anti-vibration gloves, para-aramid yarns or high-density polyethylene have also been used for the outer layer and for various types of elastic yarns for the wrist band or backhand areas [32,33,34].

Fabric thickness is an important parameter for both cushioning capacity and comfort properties. Based on the fundamental theory of mechanics where, for good vibration isolation, it is necessary to reduce the dynamic stiffness of the isolator material for low resonant frequency values during vibration, it has been deduced that the use of thicker materials is highly recommended for this purpose. In order to achieve a relatively high thickness without compromising flexibility, dexterity, and tactile sensitivity, some research has focused on achieving an optimal thickness for specific vibration isolation applications [35,36,37].

The vibration behaviour of polyester filament yarns was investigated using a device equipped with a high-speed digital camera to record the vibrations of polyester filament yarns at all of their points. Content analysis and processing of the vibration signal showed that filament orientation, yarn thickness, filament count, filament appearance (flat or textured), stimulation distance, and axial tension force affected the signal characteristics [38]. An electronic yarn with vibration detection was produced by embedding a commercially available accelerometer in the structure of a yarn. The vibration-sensitive electronic yarn was then incorporated into a fabric sample, and four vibration-sensitive electronic yarns were used to produce a vibration-sensitive glove capable of monitoring vibrations on the palm and index finger [39].

In summary, the study on spacer fabrics for anti-vibration products confirms their performance characteristics in this respect and their high potential as a substitute for PU foams. The main factors such as the test direction, yarn thickness, density, and thickness of the material can influence the value of their experimentally determined natural frequencies. It is therefore recommended that future research should employ advanced technologies to optimise their values for effective control [40,41].

## 2. Natural Frequencies and Vibration Transmissibility of Knitted Materials

### 2.1. Vibration Parameters

Vibration is generally described as a mechanical movement or oscillation around a reference point of equilibrium and is characterised by two important parameters: frequency and amplitude [2].

To characterise the mechanical behaviour of knitted fabrics, the free vibration method can be used. This means that an elastic system is brought out of stable equilibrium and generates free vibrations when released. In the presence of frictional forces, the mechanical energy is dissipated and the vibration is damped by a certain number of cycles. The frequencies of the free oscillations depend on the mass, stiffness, and damping of the system and are independent of the external forces of the system. Therefore, their frequencies are called natural frequencies of vibrations [9].

### 2.2. Vibration Transmissibility

**Figure 1:**
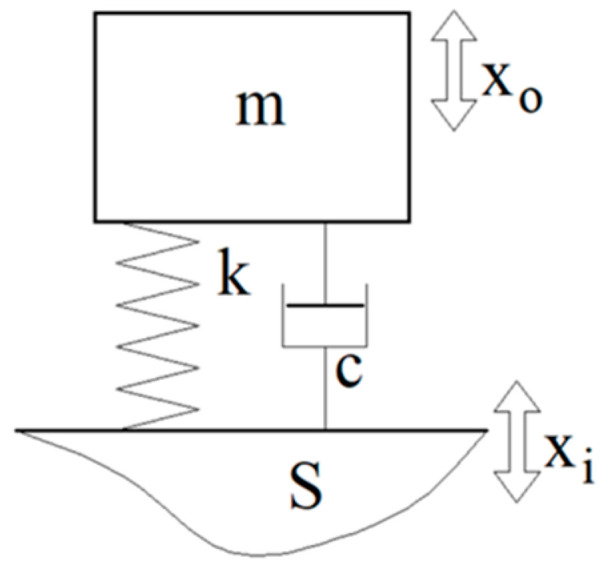
SDOF vibratory system.

The knitted fabric is characterised by internal damping (the behaviour of a damper, mainly due to internal friction) and stiffness (the behaviour of a spring). The internal damping describes the ability of a material to dissipate modal mechanical energy or to irreversibly convert the modal energy from vibration into heating. The internal stiffness describes the magnitude of the reversible deformation under the action of a compressive force. The internal stiffness is defined as the ratio between the compressive force and deformation. One of the best and simplest ways to estimate the damping and stiffness properties of a knitted fabric is to use a single-degree-of-freedom (SDOF) vibration system, a spring-mass-damper (SMD) system, placed on a mobile support S (Figure 1).

The role of the suspension of the SMD system is usually played by a spring, with the stiffness *k*; and a damper, with the damping factor *c*. In this research, the suspension has been replaced by a knitted fabric placed between the mass *m* and the support S. If the support S has a vertical harmonic vibratory motion *x_i_* = *X_i_sin*(*ωt*) as excitation, then the mass *m* also has a vertical harmonic vibratory motion *x_o_* = *X_o_sin*(*ωt* − *α*) as response, with the same excitation angular frequency *ω* = 2*πf* (with the frequency *f*) but with a different amplitude (*X*_0_ ≠ *X_i_*) and with a phase shift *α* between response and excitation. In these conditions, the SMD system works as a vibration isolator. The transmissibility *T* of the isolator, defined as *T* = *X*_0_/*X_i_*, is the ratio between the amplitudes *X*_0_ and *X_i_*. The transmissibility is a dimensionless characteristic. Some constants used to characterized the behaviour of the vibratory system are defined in the following:

- The natural undamped angular frequency: p = √(k/m);
- The damping ratio: ξ = c/2mp;
- The relative angular frequency: η = ω/p.

The vibration theory establishes [28] that the transmissibility T related by relative angular frequency is depicted as:

(1) $$T\left(\eta \right)=\frac{\sqrt{1+{\left(2\xi \eta \right)}^{2}}}{\sqrt{{\left(1-\eta^{2}\right)}^{2}+{\left(2\xi \eta \right)}^{2}}}$$

The transmissibility related by excitation frequency *f* is depicted as:

(2) $$T\left(f\right)=\frac{\sqrt{1+{\left(2\xi \frac{2\pi f}{p}\right)}^{2}}}{\sqrt{{\left[1-{\left(\frac{2\pi f}{p}\right)}^{2}\right]}^{2}+{\left(2\xi \frac{2\pi f}{p}\right)}^{2}}}$$

The evolution of the transmissibility will be experimentally used in order to find out a characterisation of the internal damping and the stiffness for different knitted fabric samples (KFSs).

**Figure 2:**
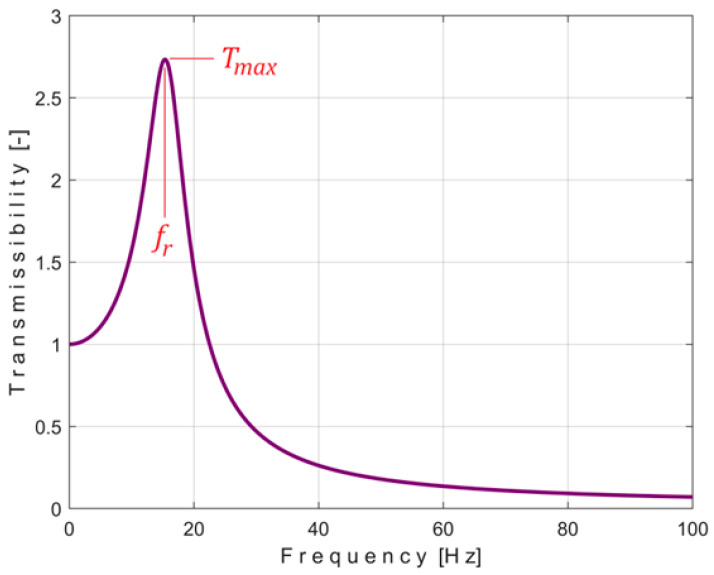
Theoretical simulation of the transmissibility.

Supposing that the transmissibility evolution *T*(*f*) is known by experimental determination, an ideal SDOF SMD system provides a transmissibility, as Figure 2 indicates, as a result of simulation. The maximum value of the transmissibility *T* happens in *T*_max_ at resonance when the condition *ω* = *p* or 2*πf_r_* = *p* = $\sqrt{k/m}$ is accomplished (the excitation frequency *f* is equal with the resonance frequency *f_r_*).

The value of resonance frequency *f_r_* can be measured from the graph and the mass *m* is available by weighing, so the stiffness *k* is available by calculating:

(3) $$k=m{\left(2\pi f_{r}\right)}^{2}$$

If *s* is the surface area of the knitted fabric sample placed between the mass *m* and the support S, then *k_s_* = *k*/*s* is the specific stiffness (the stiffness of a unit of surface area of the knitted fabric sample).

At resonance (*f* = *f_r_*), the transmissibility has a maximum value *T_max_* in Figure 2. In accordance with Equation (2), because 2*πf_r_* = *p:*

(4) $$T_{max}=T\left(f_{r}\right)=\frac{\sqrt{1+{\left(2\xi \right)}^{2}}}{\sqrt{{\left(2\xi \right)}^{2}}}$$

It follows that the damping ratio *ξ* is defined as:

(5) $$\xi =\sqrt{\frac{1}{4(T_{max}^{2}-1)}}$$

And the damping factor *c* is defined as:

(6) $$c=2m\xi p=4\pi mf_{r}\sqrt{\frac{1}{4(T_{max}^{2}-1)}}$$

If *s* is the surface area of the knitted fabric sample placed between the mass *m* and the support S, then *c_s_* = *c*/*s* is the specific damping factor (the damping of a unit of surface area of the knitted fabric sample).

In order to determine these two dynamic properties (the stiffness k and the damping factor c, or also the specific stiffness ks and the specific damping factor cs) for each knitted fabric sample, it is necessary to determine what the transmissibility curve looks like.

## 3. Materials and Methods

### 3.1. Knitted Materials

The yarns used for the manufacturing of the knitted spacer fabrics were cotton and cashmere for the outer layers and monofilaments of polyester yarns to join the layers, as shown in Table 1. The number of yarns in each feeder was chosen to match the machine gauge.

The spacer fabrics were produced on the CMS 530 E6.2 multigauge flat knitting machine from Stoll by Karl Mayer (Obertshausen, Germany). Six variants of spacer fabrics (P1-P6) were designed using Stoll M1plus^®^ pattern software (Version 7.2), one of the most effective CAD systems for creating patterns for a highly optimised knitting process. The specific tools provide optimal support for time-saving and reliable pattern creation. Different views, such as the symbol view, the fabric view, or the technical view, facilitate the realisation of complex fabrics. M1plus^®^ enables a time-saving workflow that ensures a highly optimised knitting process. For the present research, the adjusted knitting parameter was the stitch depth at three levels, by changing the position of the stitch cam, through the NP values, which can be set in the knitting program. The minimum and maximum values of the stitch depth were selected according to the knitting performance, and the fabrics were defined as tight (NP = 11.2), medium (NP = 11.5), and loose (NP = 11.8). The knitted patterns and their cross-sections as well as their description are listed in Table 2.

The combination of the previously mentioned variables, the structure, stitch cam value, NP, and yarns, has resulted in a number of 54 patterns, which are coded as follows: P1 Co1/40 NP 11.2 (pattern 1, created from cotton yarn, a 1/40 Nm gauge, with an NP 11.2 stitch cam value). After the knitting process, the fabric samples were kept under standard atmospheric conditions for the relaxation.

The two most important basic properties of the materials, the mass per unit area (g/m^2^) and the thickness (mm), were determined in accordance with the standards [42,43] and their measuring instruments are listed in Table 3.

### 3.2. Measurement of the Natural Frequencies of the Materials

**Figure 3:**
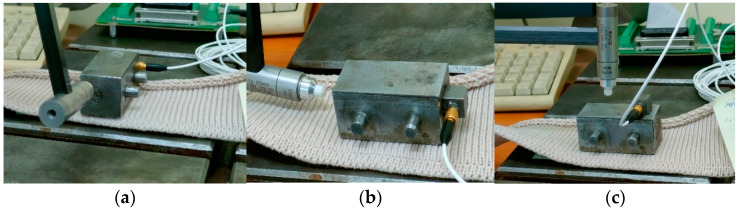
Free vibration method applied to knitted materials. (**a**) Wale-wise, (**b**) course-wise, (**c**) perpendicular.

The dynamic performance of the knitted fabrics was analysed by testing the behaviour of a piece of metal weighing 1568 g that was placed on the sample and attached to a metal mass with an adhesive to prevent its movement. The equipment used to excite the system consisted of a piezotronic impact hammer. Measurements were performed using a PCB B52 piezotronics accelerometer attached (Depew, NY, USA) to the metal mass and connected to the 6023 National [16] data acquisition board. Figure 3 shows the main components of the testing method and the testing directions of the knitted materials.

To determine the natural frequencies of the system, fast Fourier transformation (FFT) was applied and the Spectrum Analyzer application from LabView 8.2 software was used. FTT generalises the signal decomposition into a sum of sinusoids and non-periodic signals [16]. The frequencies [Hz] were determined in three directions: wale-wise, course-wise, and perpendicular on the fabric surface.

**Figure 4:**
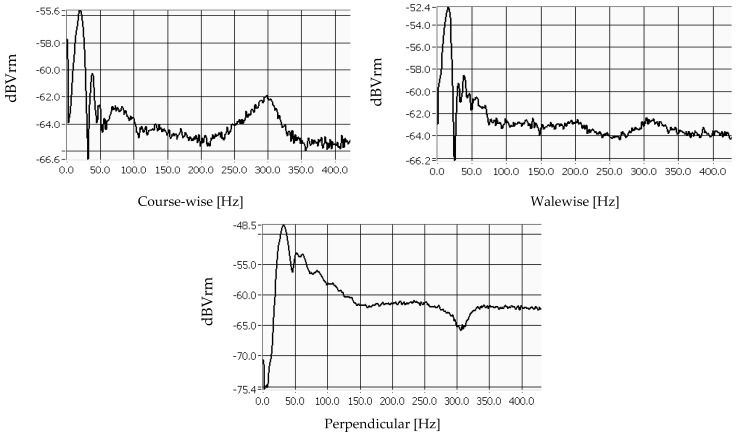
Measured natural frequencies of pattern 4, cotton Nm 1/50 yarns.

Each measurement was repeated three times, and each graph was generated by LabView software for all variants and test directions. The curves consist of waves, and the highest peak of the first wave in the horizontal direction represents the natural frequency of the material, measured in [Hz]. These values were recorded for all knitted fabrics and analysed in correlation with the most important parameters of the materials. Figure 4 shows the values for sample no. 4, which was produced with cotton yarns, Nm 1/50, and a stitch cam depth of NP 11.5.

These diagrams can be used to determine the magnitude of the natural frequency for each material, which provides information about its stiffness. The higher the natural frequency, the more stiffness that can be expected. The shape of the curve also indicates how well the sample dampens the vibrations. The smoother the shape, the higher the expected damping capacity of the material.

### 3.3. Measurement of the Vibration Transmissibility

**Figure 5:**
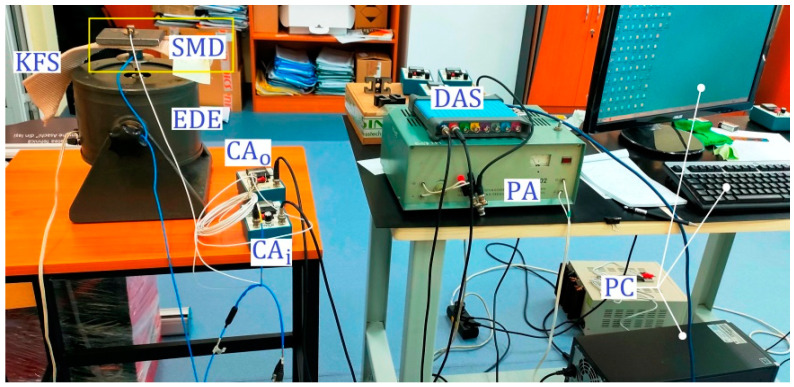
The experimental setup.

**Figure 6:**
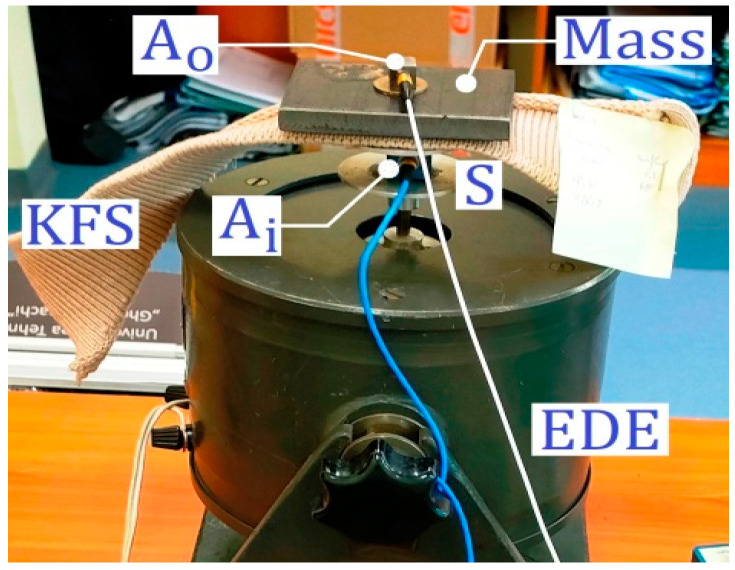
Details of the excitation system.

The experimental setup for measuring the transmissibility T is shown in Figure 5 and Figure 6, where the excitation system can also be seen. First, the SMD system is attached to the moving part of an electrodynamic exciter (EDE) with the knitted fabric serving as suspension. This movable part of the EDE plays the role of support S, which has already been introduced in Figure 1. If the EDE is properly electrically supplied (with a harmonic electrical voltage and controllable frequency), the moving part can generate a harmonic movement xi = Xisin(ωt).

For each angular excitation frequency ω or excitation frequency f (ω = 2πf), the amplitudes Xi and X_0_ are measured, and T is calculated as T = X_0_/Xi. Measuring the amplitudes Xi and X_0_.

There is an easier way to measure transmissibility: using absolute acceleration sensors (accelerometers). It is known from vibration theory that the ratio between the amplitudes of motion X_0_ and Xi is strictly equal to the ratio between the amplitudes of acceleration ωXo and ωXi (in other words: T= X_0_/Xi = ωXo/ωXi). One accelerometer (as input accelerometer Ai) is placed on the support S (or also the moving part of EDE), and the other accelerometer (as output accelerometer A_0_) is placed on the mass m (both accelerometers are fixed with beeswax, as recommended by the manufacturer). The signal supplied by each accelerometer is amplified in a charge amplifier (CAi for Ai and CAo for Ao). Each amplifier generates a voltage (a harmonic signal proportional to the acceleration). If the accelerometers and the charge amplifiers are identical, the transmissibility is simply calculated as the amplitude of the voltage generated by CAo divided by the amplitude of the voltage generated by CAi. A data acquisition system (DAS, or numerical oscilloscope) and personal computer (PC) are used for signal processing. This numerical oscilloscope has an additional function: it can generate a harmonic signal (with controllable amplitude and frequency). This harmonic signal is used to supply the EDE via a power amplifier PA [29]. An interesting function of the oscilloscope makes it possible to generate a harmonic voltage with constant amplitude and slowly linearly varying frequency in a desired range (a sinusoidal sweep signal). This voltage is applied to the power amplifier PA. The output of PA feeds EDE, which ensures the harmonic movement of xi in the vertical direction (on the moving part).

The computer-aided signal processing was carried out in Matlab (Version R2019b). First, the two signals were saved in a .txt file, and this file was loaded into Matlab software. Here, a sequence was determined for each signal for each period. The frequency f was calculated from each sequence, and the peak-to-peak voltage was determined. Half of this voltage is proportional to the amplitude of the acceleration. This procedure ensures that any offset of the signal is eliminated. The ratio between these signal halves is equal to the transmissibility T.

**Figure 7:**
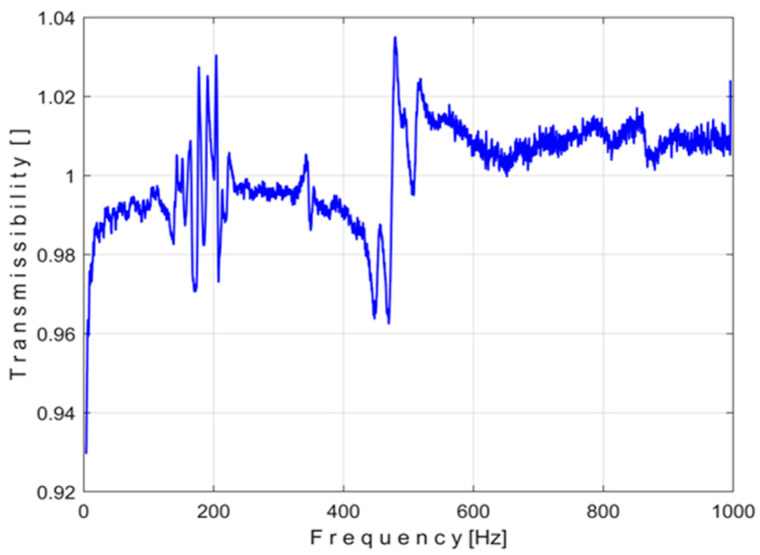
The evolution of the transmissibility.

A preliminary experiment without KFS was conducted (Figure 1, the mass is placed directly on the support S). The evolution of the transmissibility T(f) during this experiment can be graphically depicted as shown in Figure 7.

Contrary to expectations, this evolution is not a straight horizontal segment line (parallel to the x-axis at ordinate 1, the transmissibility should be equal to one regardless of the frequency value). There are some hypotheses for the deviations (however small and negligible) of the transmissibility from a straight horizontal line: the accelerometers are not placed exactly on the same vertical, the effect of some vibration modes of the accelerometer wires, etc.

## 4. Results and Discussion

### 4.1. Knitted Fabric’s Natural Frequencies

#### 4.1.1. Influence of the Test Direction on the Natural Frequencies of the Fabrics

**Figure 8:**
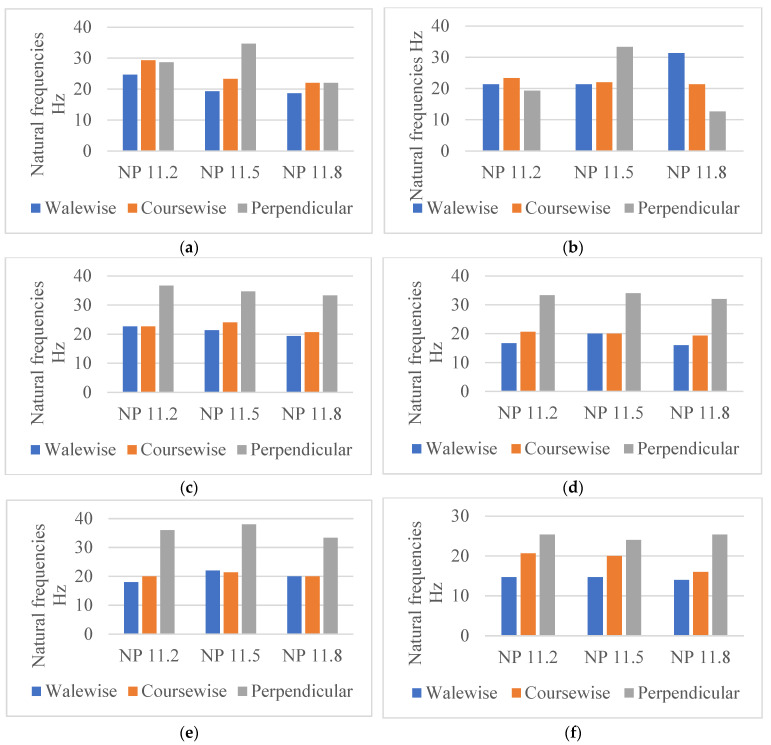
Natural frequencies measured in different directions for cotton Nm 1/40 materials. (**a**) P1, (**b**) P2, (**c**) P3, (**d**) P4, (**e**) P5, (**f**) P6.

In the present study, the knitting direction was considered to determine its influence on the natural frequency, as it has been shown to have an effect on various mechanical properties of knitted materials [16]. The experimental data of the natural frequencies for each yarn type and structure were analysed. The cotton Nm 1/40 materials and the frequency range for the three test directions are shown in the example in Figure 8a–f.

The vibration mechanism, which in this case does not involve any movement or distribution of the yarn within the structure, due to the fixed sample, is responsible for the comparable frequency level of all fabrics in the course-wise (16–29.33) Hz and wale-wise (14–31.33) Hz directions. The highest values of the natural frequencies of the knitted fabrics were measured in the perpendicular direction (12.67–38) Hz, which indicates a stronger stiffness of the material in the test direction. The shape of the frequency curve, which has only minimal peaks and a smooth profile, confirms the material’s great ability to dampen vibrations well in the perpendicular direction of the materials.

The results show comparable values of the frequencies in the wale and course directions, for all patterns and variants, approximatively within the (14–28) Hz range. Therefore, when developing a product with the fabrics being positioned in one of these directions, the external vibration system should not act in the same frequency range. The values obtained in the perpendicular direction (24–39) Hz show 30% higher measured frequencies for P1, P2, and P6 and 50% higher for P3, P4, and P5, which was expected due to the cross-sectional geometry given by the ratio of the connecting filaments and specifically for spacer fabrics.

#### 4.1.2. Influence of the Fabric’s Thickness on the Natural Frequencies

**Figure 9:**
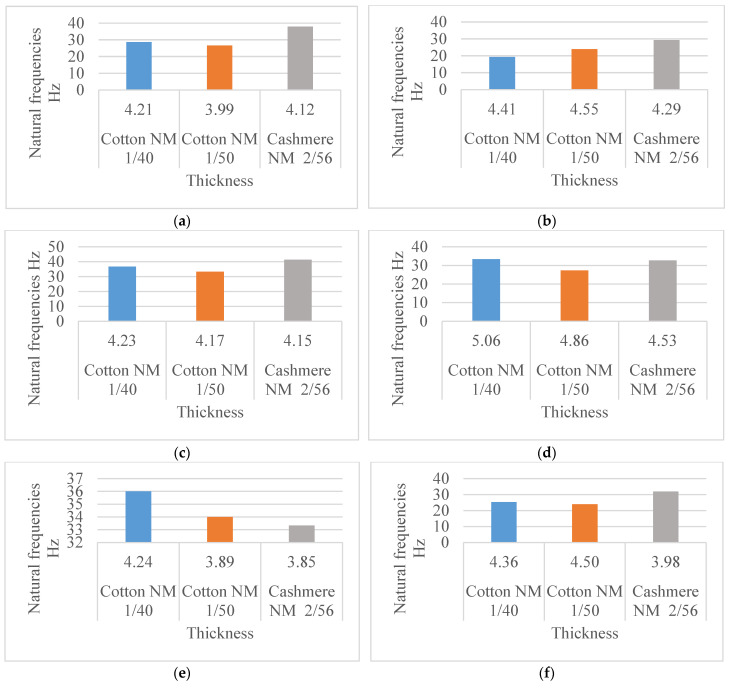
Natural frequencies in correlation with fabric thickness for fabrics produced with NP 11.2. (**a**) P1, (**b**) P2, (**c**) P3, (**d**) P4, (**e**) P5, (**f**) P6.

The fabric thickness can be the decisive parameter for fabrics with a constant thickness over the entire surface. Fabrics with higher thickness and lower stiffness are recommended for vibration isolation [29]. In Figure 9, it can be seen that when the thickness decreases, the natural frequency increases for all yarns and structures, but a different behaviour is noticeable in P1, P4, and P5, for stitch cam value NP 11.2, due to their non-uniform thickness resulting from the structural connection of the two layers, which generates a channelled structure. When more closely analysing the graphs, the recommended structures would be: P1 and P2 composed of cotton 1/40, which are thicker but less rigid than fabrics composed of cotton 1/50 and cashmere 2.56. In the case of P3, P4, P5, and P6, cotton 1/50 fabrics are the best candidates, having a considerable thickness but also a lower rigidity.

The vibration damping capacity of a material is directly related to the thickness of the material. The results show that the natural frequency of most yarns and structures increases with decreasing thickness. P3, P4, P5, and P6 patterns composed of cotton 1/50 are recommended as they have a considerable thickness but less stiffness.

The conclusion is that we can control the thickness of the fabric by the structure of the spacers, especially the choice of needles from the outer layers, and the ratio of connecting threads. If you combine this with different yarn counts, you can achieve different variations of fabrics. The ideal material for vibration isolation is that which has a greater thickness and lower stiffness [29].

#### 4.1.3. Influence of the Fabric’s Stitch Density on the Natural Frequencies

**Figure 10:**
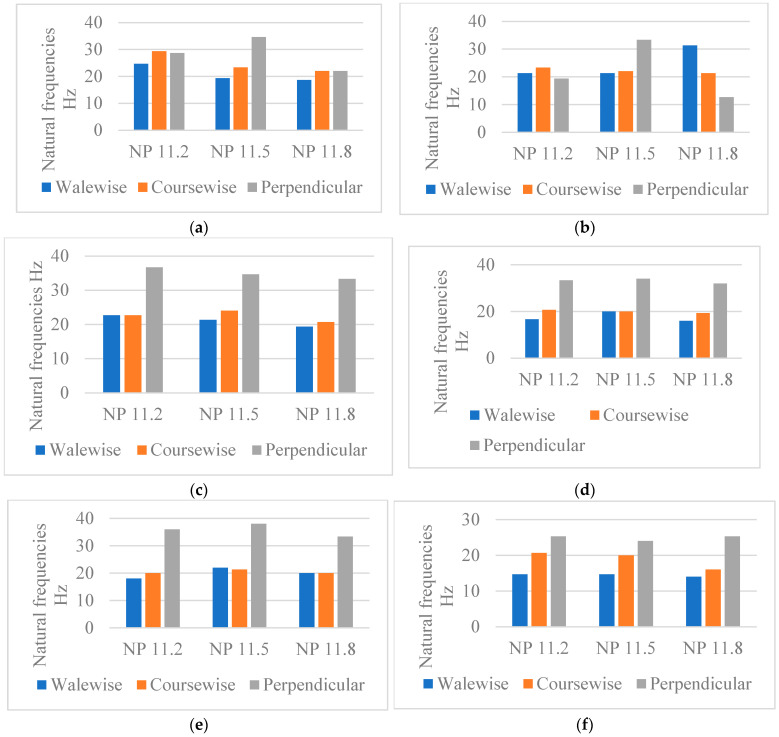
Natural frequencies of cotton Nm 1/40 materials. (**a**) P1, (**b**) P2, (**c**) P3, (**d**) P4, (**e**) P5, (**f**) P6.

**Figure 11:**
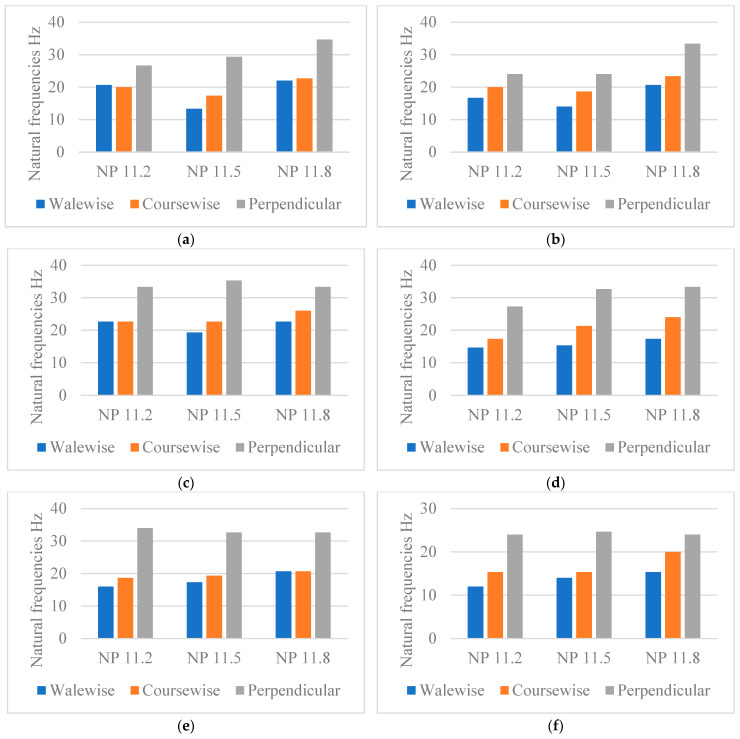
Natural frequencies of cotton Nm 1/50 materials. (**a**) P1, (**b**) P2, (**c**) P3, (**d**) P4, (**e**) P5, (**f**) P6.

**Figure 12:**
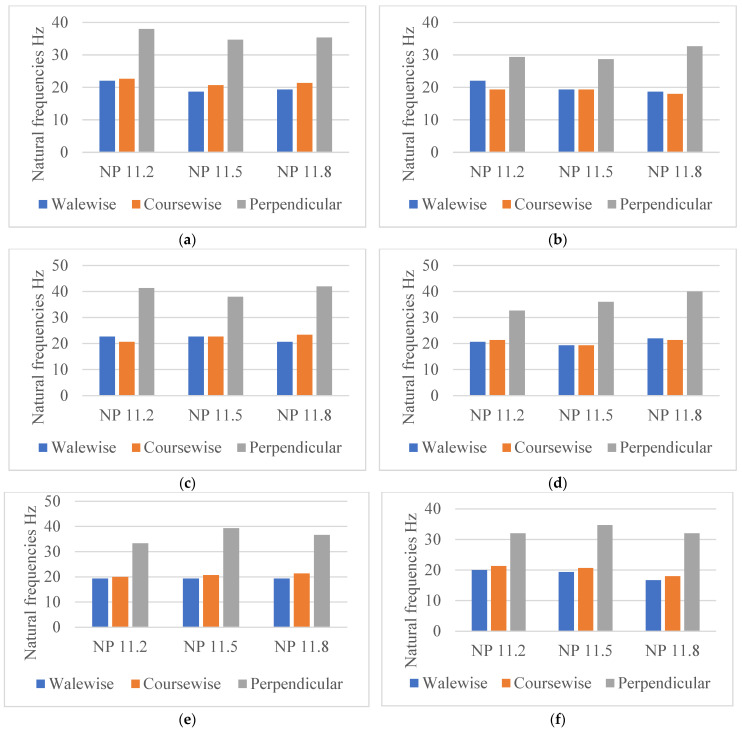
Natural frequencies of cashmere Nm 2/56 materials. (**a**) P1, (**b**) P2, (**c**) P3, (**d**) P4, (**e**) P5, (**f**) P6.

The stitch depth influences the stitch length and the density of the knitted fabric, which in turn have a considerable influence on the properties of the knitted fabric, such as dimensional stability, weight, comfort, and mechanical properties. With electronic flat knitting machines, the stitch density of the knitted fabric can be adjusted via the position of the stitch cam (NP). The higher the NP value, the longer the stitch length and the looser the fabric. For cotton Nm 1/40, cotton Nm 1/50, and cashmere Nm 2/56, this trend can be observed for all structural variants in the perpendicular direction (Figure 10, Figure 11 and Figure 12).

Previous studies have shown that the natural frequency of fabrics with the highest NP value decreases, which is reflected in a lower stiffness of the fabric [24].

Analysing the graphs, one can observe that the structures that are not following this trend are: cotton Nm 1/40: P6 NP 11.8, cotton Nm 1/50: P1 NP 11.8, P2 NP 11.8, and P4 NP 11.8; and cashmere Nm 2/56: P2 NP 11.8, P3 NP11.8, and P4 NP 11.8. An explanation for these exceptions can be that, in these programs with looser NP, the spaces between the jersey’s layers are higher, leading to increased absorption capabilities.

Due to the dispersion of needles and thus the improved damping at loose NP, pattern P6 exhibits bigger gaps than P2 in this scenario. This is because the intervals between jersey layers are closer and the damping increases as NP increases. As for P1, compared with P5, the distribution of connecting yarn on all of the needles makes this outcome reasonable because the spaces between jersey layers are small, and with increasing NP value, the spaces become bigger; thus, the damping capacity becomes stronger.

P4 is comparable with P2, with the enlargement of NP increasing the gaps in the interior and the gaps on the outer layer (one after the other), together with the elimination of the working needles compared with P2. In P3, only half of the needles are in use, as the two rows of connecting yarn are separated and one row after the other offers freer space.

#### 4.1.4. The Influence of the Yarn Type and Count on the Natural Frequencies

**Figure 13:**
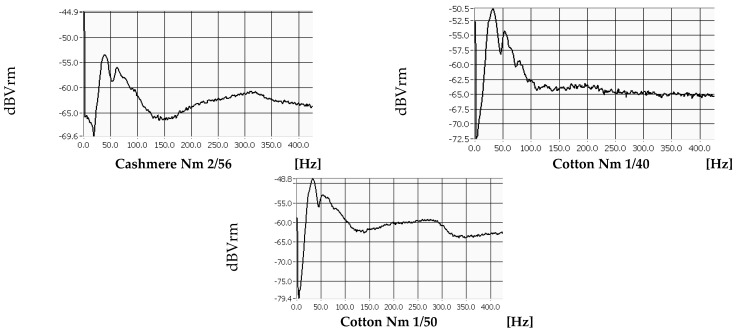
Natural frequencies of P4 in the perpendicular direction.

The yarn count affects the response of the fabrics in the dynamic tests. An analysis of experimental data selected for all fabrics in the perpendicular test direction shown in Figure 13 elucidated that, as expected, there is no significant difference between cotton Nm 1/40 and cotton Nm 1/50 but a noticeable difference between cashmere Nm 2/56 and the two yarns of cotton Nm 1/40 and Nm 1/50.

This tendency does not apply to the samples created from cotton Nm 1/40: P1 NP 11.5, P2 NP 11.2, P5 NP 11.5; and the samples created from cotton Nm 1/50: P1 and P2 NP 11.8. This can be explained by two factors:

- Firstly, it is the characteristics of each of these yarns, such as the count of the yarn and the twisting process, that make up the difference between cashmere and cotton, with the section of cashmere yarn being larger due to the twisting process, unlike cotton yarns where the two yarns are fed into the yarn feeder parallel to each other;
- Secondly, the distribution of the connecting yarn in these programs plays a factor, which influences the distances between the two outer layers depending on the NP.

As already mentioned, the shape of the curve in the natural frequency diagrams shows how well the material can absorb vibrations. The cashmere fabric absorbs the vibrations better than the cotton fabric because its shape is the smoothest compared with the cotton fabric in the perpendicular direction, as can be seen in Figure 13.

**Figure 14:**
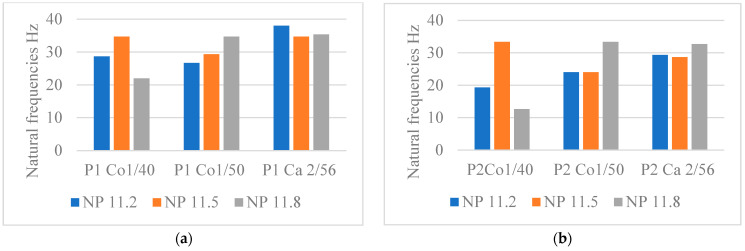

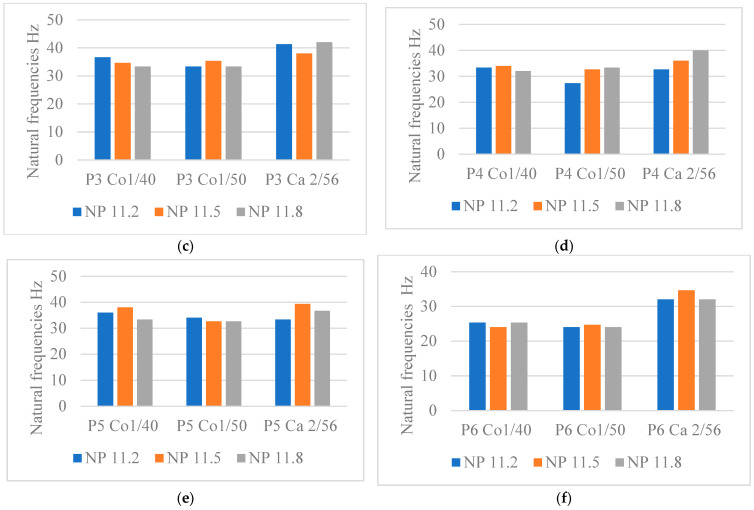
Influence of the yarn account on the natural frequencies of knitted structures. (**a**) P1, (**b**) P2, (**c**) P3, (**d**) P4, (**e**) P5, (**f**) P6.

As can be seen in Figure 14, knitted cashmere has the highest natural frequency at NP11.8. This is due to the fact that cashmere Nm 2/56 is the finest yarn but has the largest yarn cross-section compared with the other yarns, which is created by the twisting process. It is reasonable to assume that the raw materials used can control the natural frequencies of textiles for a specific knitted fabric in the design phase.

### 4.2. Knitted Fabric’s Vibration’s Transmissibility

**Figure 15:**
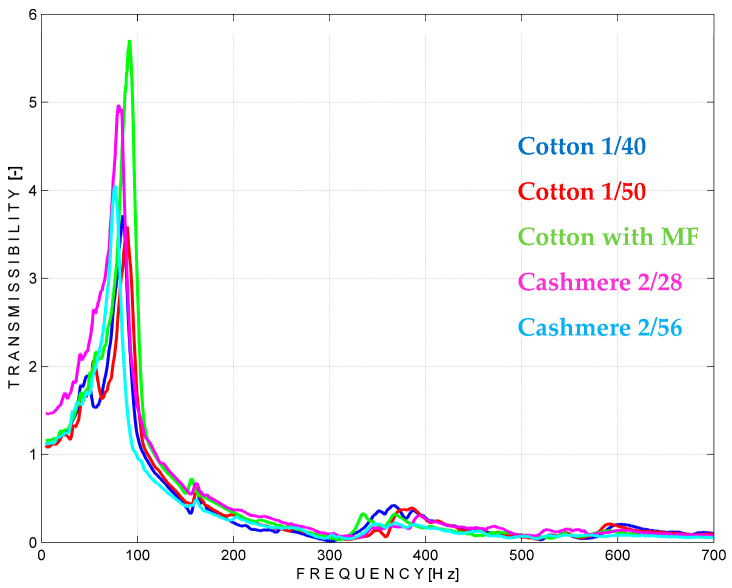
Vibration transmissibility simulation on the left part of the materials.

According to the vibration theory and the experimental approach explained in Section 2.2 and Section 3.3, respectively, several knitting patterns with rectangular L > l shape were tested by performing three sets of measurements. The first set refers to the use of the left zones of the material located between the mass and the support S. The results, in terms of vibration transmissibility, are shown in Figure 15. In the graphs, three raw materials presented in the previous sections, cotton Nm 1/40 and Nm 1/50 and cashmere Nm 2/56, can be identified.

The simulation curves can be used as a guide to characterise the knitted materials in terms of transmissibility of the vibrations. For example, the lowest transmissibility shows cotton Nm 1/50 and cotton Nm 1/40, followed by cashmere Nm 2/56. This property can be used in both directions, either to allow the material to transmit the vibration, i.e., in the case of a massage product, or to act as a barrier against vibration when it comes to protective purposes. From the graphs, the frequency range of the external system in which these materials can isolate best can be determined. In this case, all tested materials show the lowest values for transmissibility in an environment with a frequency of 300–400 Hz. They can also show good insulation properties in the 500–700 Hz range, as shown in the diagrams. Conversely, when used for the transmission of vibrations, they can facilitate the transmission of vibrations in the 100 Hz range of the external system. The experimental data have been used to calculate the specific stiffness and specific damping according to the formulas detailed at Section 2.2, and the results are given in Table 4.

The values shown in Table 4 confirm the previous analysis regarding the transmissibility of the materials, with the highest specific damping coefficient belonging to fabrics composed of cotton Nm 1/50 and the lowest belonging to those composed of cashmere Nm 2/56. This information is useful if you are designing a product for a specific use that has special requirements for the environmental frequencies from which it must be protected.

**Figure 16:**
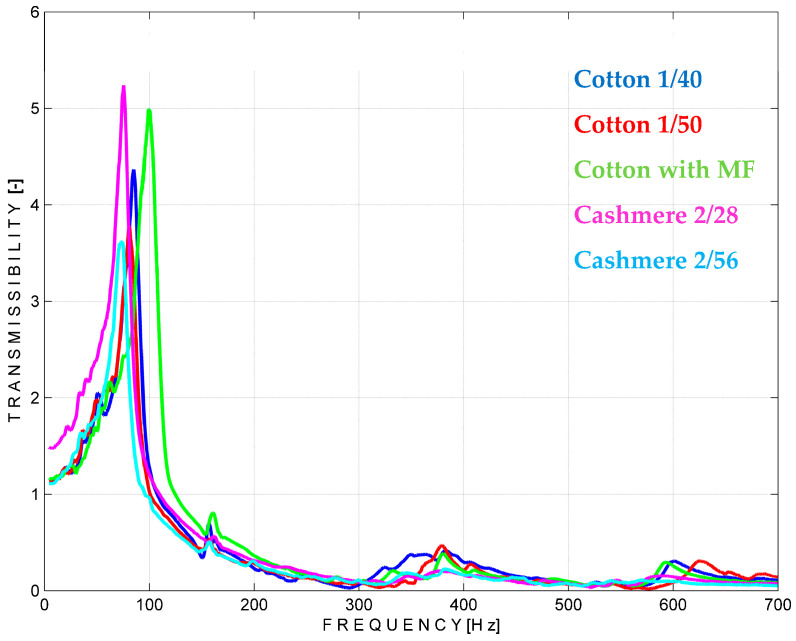
Vibration transmissibility simulation on the middle part of the materials.

**Figure 17:**
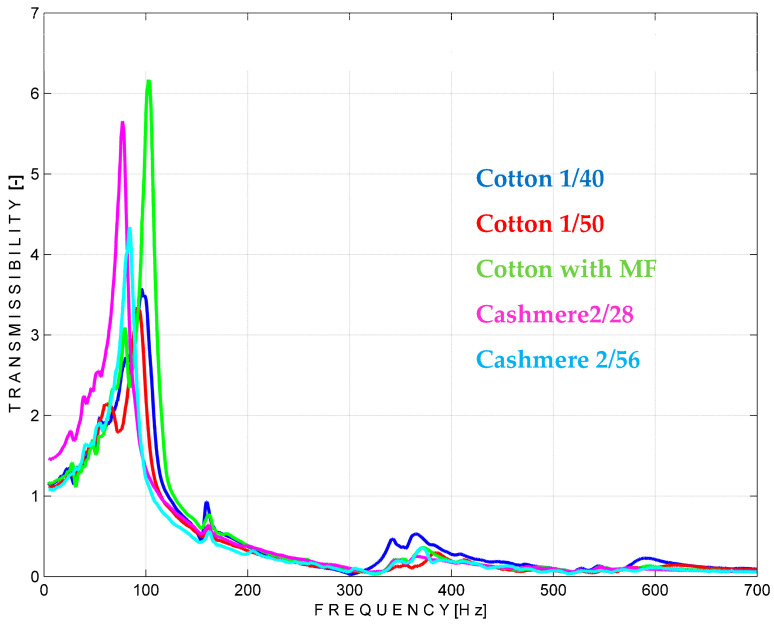
Vibration transmissibility simulation on the right part of the materials.

A closer look at the measurements of the middle and right parts of the materials from Figure 16 and Figure 17 and the calculated parameters from Table 5 and Table 6 shows some differences. These could be explained by the structural range of the measurements, which could be different in the thickness and distribution of the yarns due to the particular way in which the yarns of the outer layers are joined together.

The specific vibration parameters are calculated according to the formulas detailed at Section 2.2 and the results are given in Table 5 for the middle part of the tested fabrics.

## 5. Conclusions

One of the key elements in reducing vibrations is the ability to produce special protective materials. The use of spacer knitted fabrics with vibration damping properties is a promising research direction as the technology to develop and manufacture these materials with suitable, repeatable physical properties such as weight, thickness, stitch density and type of raw materials is advanced [20].

The natural frequencies of spacer knitted fabrics produced on electronic flat knitting machines were measured in three directions using the free vibration method. The values obtained in the perpendicular direction show 30% higher measured frequencies for P1, P2, and P6 and 50% higher for P3, P4, and P5 compared with the wale-wise and course-wise directions, which is due to the cross-sectional geometry given by the ratio of the connecting yarns and especially for spacer fabrics.

The thickness of the fabric, as a key parameter of the spacer knitted materials, can be adjusted through the needle’s selection in the outer layers and the ratio of connecting threads, with fabrics with higher thickness and lower stiffness being recommended for vibration isolation. The results show that the natural frequency increases with decreasing thickness for all patterns. P3, P4, P5, and P6 patterns composed of cotton 1/50 are recommended as they have a considerable thickness but less stiffness.

The knitted materials vibration transmissibility has been tested using a single-degree-of-freedom vibration system, and the results show that cashmere materials have the highest transmissibility and the lowest specific damping coefficient. The materials created from a cotton Nm 1/50 yarn count exhibited the lowest transmissibility and the best insulation properties, as evidenced by the highest value of the damping coefficients.

This research can serve as a background for future studies on the development of new textile materials that can serve either as insulators or as transmitters of vibrations. The development of new intelligent vibrating materials for medical applications is considered a future research goal [44,45]. It has been shown that WBV can have a positive effect in the medical field as vibration therapy for cancer-related bone diseases [46].

## Figures and Tables

**Table 1:** Yarns used in the knitted spacers.

Yarns	Yarn Count and Diameter	Number of Yarns per Feeder	Characteristics
Cotton	Nm 1/40	2	High breathability; good moisture-wicking properties; very susceptible to pilling; very comfortable; hypoallergenic; odour-free; easy care.
	Nm 1/50	2	
Cashmere	Nm 2/56	1	High breathability; good moisture wicking; high tendency to pilling; soft feel on the skin; good protection against low temperatures.
Polyester	Ø 0.08 mm	2	Strong and elastic; can be blended with other fibres; high moisture-wicking capacity; high flame resistance; hydrophobic.

**Table 2:** Knitted patterns produced on a Stoll CMS 530 E6.2 machine.

Knitted Patterns	Knitted Sections	Structure Description	Fabric Appearance
P1	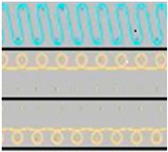	Two single jersey layers joined by a single row of connecting yarn working with all needles.	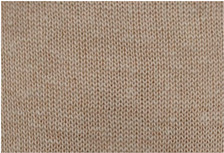
P2	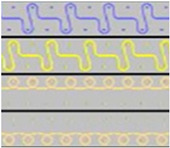	Two single jersey layers joined by two rows of connecting yarns, 1:1 successively knitted.	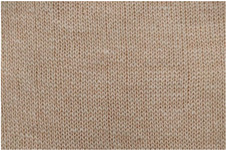
P3	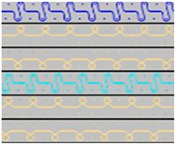	Four single jersey rows, 1:1 successively knitted, connected by two rows of connecting yarns, 1:1 successively knitted.	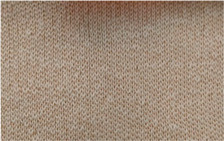
P4	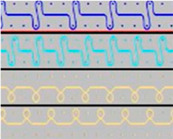	Two 1:1 single jersey rows are joined by two rows of connecting yarns, 1:1 needles are working, respectively, and 1:2 needles are working.	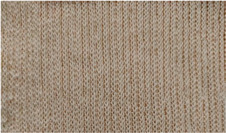
P5	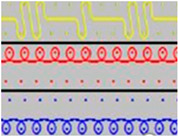	Two single jersey layers are connected together by a single row of connecting yarns, working with 2:2 needles selection.	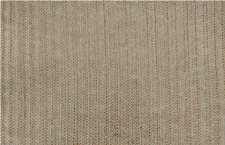
P6	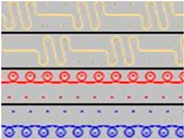	Two single jersey layers are connected together by two rows of connecting yarns, working with 2:2 needles selection.	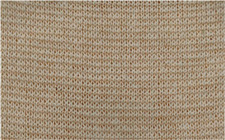

**Table 3:** Basic properties of the knitted fabrics.

Parameters	Measuring Device
Mass per unit area	Scale
Thickness	SDL Atlas M 034A digital thickness gauge

**Table 4:** Vibration parameters calculated for the left part of the fabrics.

Specific Vibration Parameters	Cashmere 2/56	Cotton 1/40	Cotton 1/50
Damping constant *c* [N·s/m]	73.7128	89.5588	97.4675
Specific damping c_s_ [N·s/m/cm^2^]	0.6203	0.7537	0.8202
Stiffness of the material *k* [N/m]	139,601.5822	172,334.259	189,992.2025
Specific stiffness *k_s_* [N/m/cm^2^]	1174.8711	1450.3456	1598.9529

**Table 5:** Vibration parameters calculated for the middle part of the fabrics.

Specific Vibration Parameters	Cashmere 2/56	Cotton 1/40	Cotton 1/50
Damping constant *c* [N·s/m]	79.856	76.1949	83.5349
Specific damping c_s_ [N·s/m/cm^2^]	0.672	0.6412	0.703
Stiffness of the material *k* [N/m]	129,456.085	173,772.866	155,537.3860
Specific stiffness *k_s_* [N/m/cm^2^]	1089.4877	1462.4528	1308.9850

**Table 6:** Vibration parameters calculated for the right part of the fabrics.

Specific Vibration Parameters	Cashmere Nm 2/56	Cotton Nm 1/40	Cotton Nm 1/50
Damping constant *c* [N·s/m]	75.3829	104.8067	107.8516
Specific damping c_s_ [N·s/m/cm^2^]	0.6344	0.882	0.9076
Stiffness of the material *k* [N/m]	168,054.3158	218,093.623	200,690.3161
Specific stiffness *k_s_* [N/m/cm^2^]	1414.3261	1835.4512	1688.9870

## Data Availability

The original contributions presented in this study are included in the article. Further inquiries can be directed to the corresponding author.
